# Use of research electronic data capture (REDCap) in a COVID-19 randomized controlled trial: a practical example

**DOI:** 10.1186/s12874-021-01362-2

**Published:** 2021-08-21

**Authors:** Sina Kianersi, Maya Luetke, Christina Ludema, Alexander Valenzuela, Molly Rosenberg

**Affiliations:** 1 Department of Epidemiology and Biostatistics, Indiana University School of Public Health-Bloomington, Bloomington, IN USA; 2 Associate Application Administrator, REDCap, Advanced Biomedical IT Core, UITS Research Technologies, Indiana University, Bloomington, IN USA

**Keywords:** REDCap, RCT, Randomized controlled trials, Randomization, Risk of bias

## Abstract

**Background:**

Randomized controlled trials (RCT) are considered the ideal design for evaluating the efficacy of interventions. However, conducting a successful RCT has technological and logistical challenges. Defects in randomization processes (e.g., allocation sequence concealment) and flawed masking could bias an RCT’s findings. Moreover, investigators need to address other logistics common to all study designs, such as study invitations, eligibility screening, consenting procedure, and data confidentiality protocols. Research Electronic Data Capture (REDCap) is a secure, browser-based web application widely used by researchers for survey data collection. REDCap offers unique features that can be used to conduct rigorous RCTs.

**Methods:**

In September and November 2020, we conducted a parallel group RCT among Indiana University Bloomington (IUB) undergraduate students to understand if receiving the results of a SARS-CoV-2 antibody test changed the students’ self-reported protective behavior against coronavirus disease 2019 (COVID-19). In the current report, we discuss how we used REDCap to conduct the different components of this RCT. We further share our REDCap project XML file and instructional videos that investigators can use when designing and conducting their RCTs.

**Results:**

We reported on the different features that REDCap offers to complete various parts of a large RCT, including sending study invitations and recruitment, eligibility screening, consenting procedures, lab visit appointment and reminders, data collection and confidentiality, randomization, blinding of treatment arm assignment, returning test results, and follow-up surveys.

**Conclusions:**

REDCap offers powerful tools for longitudinal data collection and conduct of rigorous and successful RCTs. Investigators can make use of this electronic data capturing system to successfully complete their RCTs.

**Trial registration:**

The RCT was prospectively (before completing data collection) registered at ClinicalTrials.gov; registration number: NCT04620798, date of registration: November 9, 2020.

**Supplementary Information:**

The online version contains supplementary material available at 10.1186/s12874-021-01362-2.

## Background

Research Electronic Data Capture (REDCap) is a secure, browser-based web application available in 141 countries and to around 1.6 million users [1]. The application is primarily used for developing, maintaining, and managing different types of surveys and securing online/offline data collection [2, 3]. However, REDCap offers other useful features that are less commonly used by researchers but could be valuable for conducting randomized controlled trials (RCTs).

RCTs are considered the gold-standard design for evaluating the efficacy of an intervention, specifically because they can remove most of the possible confounding bias, which is an inherent challenge for observational study designs. However, RCTs are not immune to biases [4], and like other study designs, conducting an RCT can be challenging. Managing participant recruitment, consent procedures, data collection, and data security can be time-consuming for investigators, in addition to the thoughtful consideration needed to address any barriers to participation [5]. Moreover, RCTs are susceptible to flaws in randomization and blinding processes [6].

Participation rates in human studies have been substantially decreasing over the years and innovations in study recruitment and participant retention techniques are needed to improve these rates [7]. Development of a systematic recruitment mechanism and implementing a follow-up method that ensures maximum retention rates are imperative principals for reaching to and maintaining target sample size [8]. Further, clear and detailed reporting of different measures for participation rates, including, response, cooperation, refusal, and contact rates, are important when publishing the results of a study [7, 9]. These measures are commonly missed in study reports. An automated process that can systematically record these numbers could help to overcome the mentioned challenges.

Providing concise, comprehensive, and comprehensible study details in consent forms is another ethical, legal, and essential component of any study design with human participants [10]. Participants’ comprehension of study procedures is commonly flawed [11, 12]. For instance, only 54% of studies included in a systematic review reported that their participants had adequate understanding of the studies’ aims [12]. Consent processes that are based on interactive computer programs and multimedia tools (i.e., eConsent process) could potentially improve participant comprehension of study aims, risks, benefits, and procedures [13]. Additionally, a comprehensive eConsent process could help to improve recruitment and retention rates in clinical studies [14].

Erroneous data entry (i.e., a source of measurement error) and missing data are common sources of bias in RCT study designs [15]. These are more likely to occur when using traditional data collection methods, compared to electronic data capturing tools [16, 17]. Additionally, even though researchers can use analytic approaches to deal with missing data, preventing missing data at data collection phase is a more favorable approach because the analytical approaches rely on uncheckable assumptions [15]. Electronic data capturing tools can help to minimize data cleaning and processing efforts, reduce data collection costs, enable real-time data monitoring, improve data quality, and corroborate data security [16, 18–20].

Randomization, where participants are randomly assigned to one of the intervention or control groups, is a key methodological step in an RCT because it minimizes the potential for measured or unmeasured confounders to influence study results [21]. There are two key randomization steps, sequence generation and allocation sequence concealment [21]. 1) *Sequence generation* is the process of creating a random order that determines how participants will be assigned to different groups in the RCT [21]. Defects in completing the sequence generation process have been found in many RCT study reports [21, 22]. 2) *Allocation sequence concealment* happens at the beginning of the RCT, following sequence generation, and implies that neither participants nor members of the research team are aware of the generated sequence until after the participants are assigned to their groups [23]. Like sequence generation, deviations from the allocation sequence concealment process may introduce bias to the effect estimate [24, 25].

Masking occurs after randomization and refers to the process of blinding participants and/or study staff from participants’ allocated groups [6]. Masking participants, data collectors, health care providers, investigators, outcome assessors, or other study staff members through the course of an RCT can help to reduce bias because it can prevent potential deviations from the study protocol [4, 6]. Randomization reduces confounding and selection bias and masking minimizes ascertainment bias [6]. Effect estimates from RCTs that fail to mask, or fail to fully mask, study participants and staff may lead to biases that tend to exaggerate the true effect value [22, 24, 26]. Masking can be challenging, particularly in large RCTs where there are multiple roles involved.

*Objective*: REDCap offers features that can help to address and reduce the aforementioned biases and challenges. In this study, our objective was to discuss how we used REDCap to conduct the different components of our RCT: invitation, screening, recruitment, obtaining informed consent, randomization, blinding, and data collection. The RCT was prospectively registered at clinicaltrials.gov, registration number: NCT04620798. Results of the RCT will be published in a separate report.

## Methods

### Study description

In September and November 2020, we conducted a parallel-group two-month-long longitudinal RCT among Indiana University Bloomington (IUB) undergraduate students to understand whether receiving the results of a SARS-CoV-2 antibody test changed the students’ self-reported protective behavior against this infection (e.g., physical distancing and mask-wearing). We sent study invitations and invitation reminders to all sampled students. Interested students completed the eligibility screening survey, e-signed the consent form, and scheduled an in-person antibody test appointment. After taking the antibody test, one group of participants received their antibody test results within hours of their test while the other group received their results after four weeks. All participants self-reported their level of adherence to protective behaviors at baseline and approximately every two weeks after baseline. We collected the RCT study data using REDCap electronic data capture tools hosted at Indiana University [2, 3]. Further details have been previously reported elsewhere [27, 28]. In the following paragraphs, we explain how we used REDCap in each step of the RCT.

We are sharing the metadata of our entire REDCap project, including the instruments, fields, and project attributes (Additional file 1: REDCap XML file). Interested readers can use this file to reproduce the REDCap project that we used for our RCT study. Moreover, we have made brief instructional videos for the specific REDCap features that we have introduced in this manuscript (See Supplementary Information). See Additional file 2 for a video about data collection procedures (Additional file 2: Data Collection Procedures).



**Additional file 2.**



## Results

### Study invitation, eligibility screening, and consenting procedures

#### Data import tool

In our RCT, the sampling frame was the complete list of all IUB undergraduate students enrolled in the fall semester of 2020. Initially, IUB provided a random sample of 2500 students. Later in the project, to meet our target sample size of 1700 participants, we requested an additional sample of 5000 students. Thus, a total of 7499 students were randomly sampled for the study (one of the sampled students was a duplicate from the initial sample). The sample information came in a CSV file format that included columns for students’ full name and their email address. We used the Data Import Tool to import the sample file into our REDCap project (Additional file 3: Data Import Tool).



**Additional file 3.**



#### Survey distribution tools

**Fig. 1:**
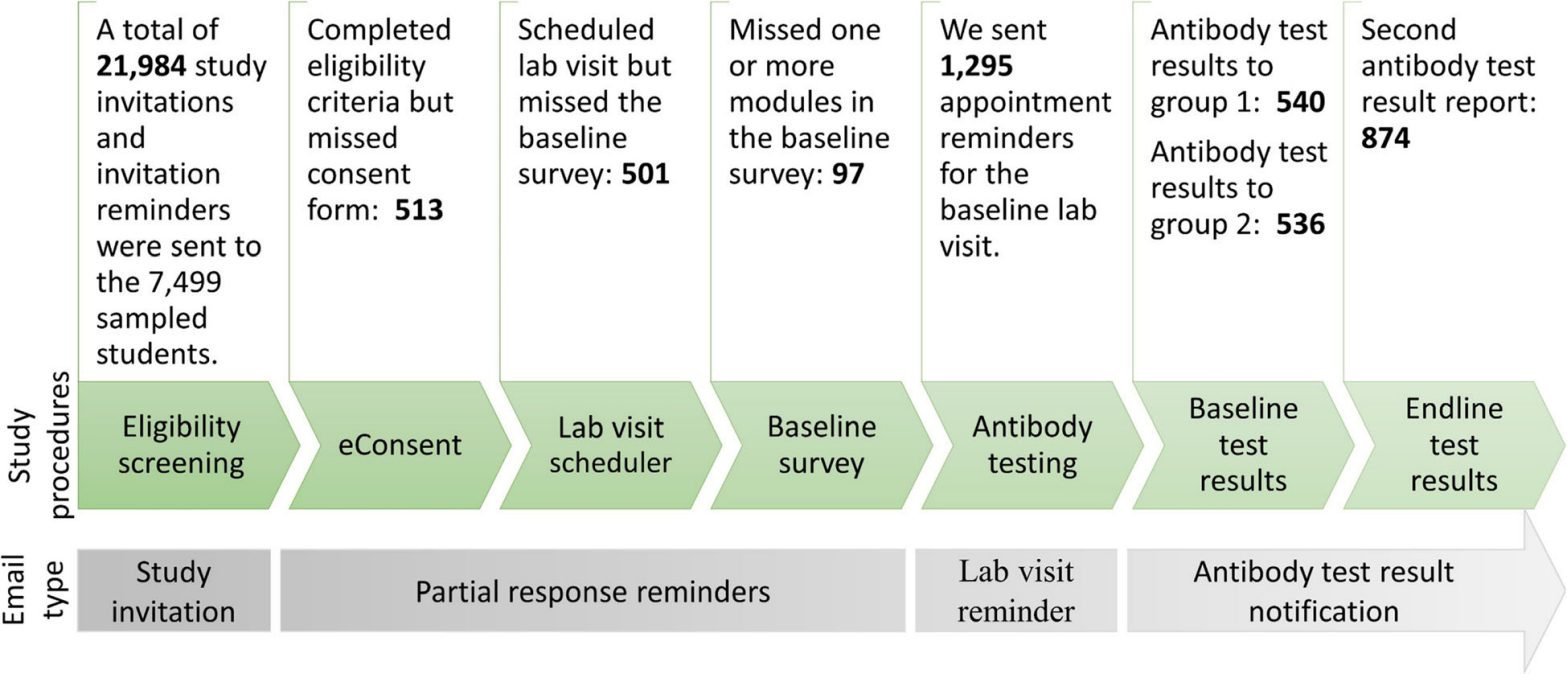
Type and number of emails sent to the study sample, identified by Survey Distribution Tools on REDCap

**Fig. 2:**
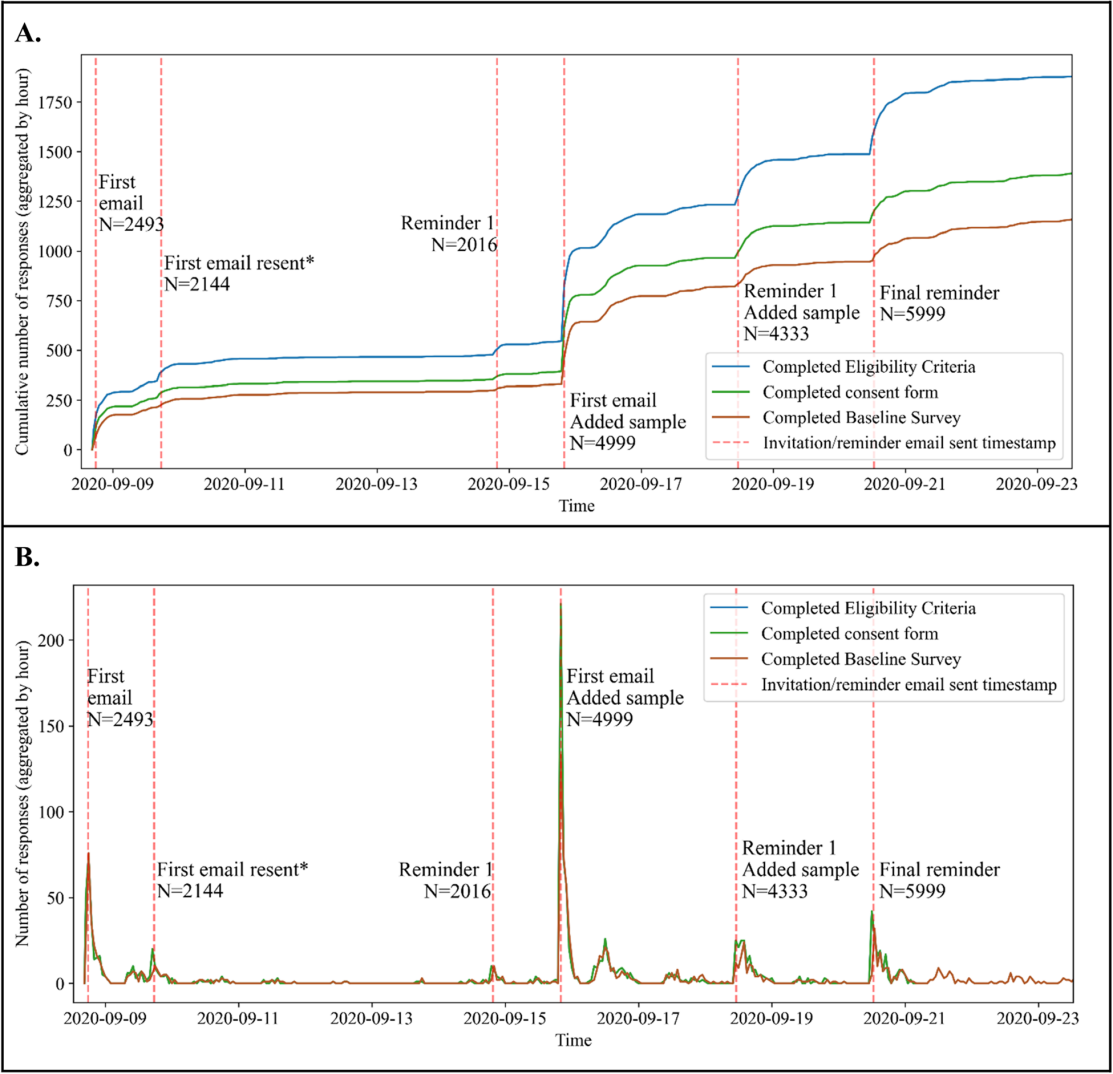
Time and number of study invitation emails sent with REDCap Survey Distribution Tools. Panel **A**. Cumulative number of responses to study invitation emails. Panel **B**. Number of responses to study invitation emails. * We resent the first study invitation to a portion of our initial sample because a subject line was omitted from the original email

We used REDCap Survey Distribution Tools and features available in the Participant List tab to send the study invitation and invitation reminder, partial response reminder email, appointment (lab visit) reminder, and antibody test result notification to our sample and keep track of their response status (Fig. 1). In total, we sent 26,340 emails to our study sample over the course of the RCT study. We sent 9636 study invitations, 6349 first study invitation reminders, and 5999 final study invitation reminders (Fig. 2). When sending the study invitation emails to our initial sample, we accidentally omitted the subject line in the email invitations. Therefore, we added the missing subject line and resent the first invitation email to the students who had not yet responded to the subject-less invitation email.

The initial invitation email included a participant-specific URL that linked to a short survey about eligibility criteria, an online consent form, lab visit scheduler, and a baseline survey. We used Compose Survey Invitation and HTML codes to design these invitation emails. Piping is a feature in REDCap that enables users to insert previously collected data into other parts of a survey or REDCap project. We used piping to display the participant’s name and specific survey link in the study invitation email. Eligible participants who consented to participate in the study were able to schedule a lab visit appointment and complete the baseline survey about various COVID-19 risk behaviors (Fig. 1, Additional file 4: Survey Distribution Tools).



**Additional file 4.**



Though not yet widely publicized by our REDCap instance at the time of this study, REDCap has useful features which enable users to invite participants through text messages and automated voice calls using Twilio, a third-party web service. Investigators can now distribute their study invitations via email, text messages, and voice calls. Inviting participants through different modes may increase the participation rate [29].

#### Survey invitation log

REDCap’s Survey Invitation Log keeps a log of sent emails, the time of distribution, the survey link included in the email, and whether participants completed the survey. Users have the option to export the log data as a CSV file for analysis. In our study, we used this file to capture the response patterns of our study sample and adapt the sending time according to temporal patterns, observed participant response rate, and appointment adherence. For instance, we noticed that the number of missed appointments was smaller when we sent the appointment reminders in the morning of the appointment day, as contrasted with sending them the night before. Sending appointment reminders to students just before they start their day appeared to remind them of their scheduled appointment. Investigators can use this feature to monitor the response patterns of their study sample in real-time and adjust the time of email distribution or the email content to improve response rates. Further, we used these log data when calculating different measures for participation rates (Additional file 5: Survey Invitation Log).



**Additional file 5.**



#### Unsubscribe survey

We added an unsubscribe hyperlink to the study invitation email so uninterested students could opt-out of receiving future reminders about the study with one click as well as provide optional information to us about their reasons for refusal. Adding this option is helpful to track non-response and the reasons for participation refusal (Additional file 6: Unsubscribe Survey). Moreover, researchers can use this technique to collect demographic data on non-responders and refusals to later assess nonresponse bias [7].



**Additional file 6.**



#### Consent form

REDCap offers an eConsent framework with various features, such as video library, wet signiture, avatar, in-line descriptive popup, analytics module, and PDF-consent document repository [14]. After obtaining approval from the Human Subjects Office about our study’s online consenting procedure, we used REDCap to create the eConsent form. We included the consent statement in a consent survey form as a Descriptive Text field and added Signature and Date fields to obtain electronic informed consent from participants. REDCap keeps records of all the signed consent forms as PDF files (Additional file 7: Consent Form).



**Additional file 7.**



### Lab visit scheduler and appointment reminders

We creatively used standard REDCap functionality to make a scheduler for in-person antibody tests. We used a Multiple Choice Drop-down List (Single Answer) field with our available dates as answer options. In REDCap, action tags are terms that start with the @ sign and can be used to control the way questions and responses are displayed for respondents. We used an action tag (@MAXCHOICE) to make a time slot disappear when it reached full capacity. For example, our nursing staff could conduct 15 antibody tests between 1:00 pm and 1:30 pm on the testing days. By setting the @MAXCHOICE action tag to ‘15’ for that time, we prevented additional appointments beyond our capacity. To make it easy for our participants to find the research site, we uploaded a map of the location to Google Drive, made the link to the map public, and shared the link along with the scheduler instrument (Additional file 8: Lab Visit Scheduler).



**Additional file 8.**



As noted above, REDCap can be used for mass email distribution. We made use of this feature when sending lab visit appointment reminders to participants. As with recruitment, we used the Survey Distribution Tools for sending the appointment reminders. We used REDCap’s piping feature to pull the participant’s name, study ID, and appointment time into the reminder emails. No survey links were included in these emails, as they were simply reminders about the participant’s upcoming antibody test appointment. These reminders were sent to all participants with a scheduled appointment (Additional file 4).

### Data collection and confidentiality

Data collection using surveys is a key function of REDCap. In our study, demographic and behavioral data were self-reported in online REDCap surveys at baseline and at four follow-up timepoints. At the in-person study visits, we used a REDCap instrument for capturing antibody test results. Trained field staff read the test results directly from the test kits and entered the data into the REDCap instrument using tablets at the study site. We used Data Exports, Reports, and Stats feature to make reports of specific instruments and events during the data collection for quality control purposes; we also used this feature when exporting the whole dataset as a CSV file. Additionally, we protected the confidentiality and privacy of participants using several data safety and protection abilities of REDCap servers. Specifically, Identifier tags kept a level of de-identification of data for in-person lab staff and the User Rights features helped us restrict access to the personal information of participants from the field staff, who did not need such data to enter in the antibody test results. Lastly, through the Shared Data Instrument Library, REDCap offers validated data collection instruments that researchers can use in their studies [30].

#### Identifier tag

It is possible to de-identify the dataset and remove protected health information (PHI) from the data when exporting the collected dataset using the Data Export tool. As a data safety measure, we used the Identifier tag on the Edit Field window in REDCap’s Online Designer to de-identify the data. This tool helped us to tag the PHI variables in our dataset and ensure that they cannot be downloaded by unauthorized users (Additional file 9: Identifier Tag and User Rights).



**Additional file 9.**



#### User rights

We used the REDCap User Rights feature to manage study staff access to parts of the project. For instance, in REDCap, staff responsible for data entry of test results were granted access only to participants’ study IDs and the instrument for entering test results. The study ID for each participant was a 7-digit unique combination of numbers and letters based on elements of participant data (e.g., certain digits of cell phone number, month of birth, etc.) that they had entered to REDCap in the online baseline survey. When entering the antibody test results, field staff used this study ID as opposed to any personally identifiable information. Field staff only had access to study IDs and did not have access to other variables or personal details (Additional file 9).

### Randomization

Sequence generation: In our RCT, we used a stratified block randomization technique to obtain an equal number of participants in the study groups (i.e., RCT arms) between those who tested positive and those who tested negative for SARS-CoV-2 antibodies. An independent statistician used SAS 9.4 (Cary, NC) and generated a random sequence (*n* = 3000) in excess of the total number of anticipated participants to account for any potential participants who might use up allocations but not continue in the study; for example, a participant who would be randomized but later withdraw from the study. REDCap provides tools for allocating treatments to participants based on the allocation sequence.

Allocation sequence concealment: Perhaps one of the most important, yet underappreciated REDCap features for conducting RCTs is its functionality for achieving allocation concealment, that is “preventing the next assignment in the clinical trial from being known” [6]. In our study, for instance, if participants had known that they were going to receive their antibody test results in 4 weeks, they might have withdrawn from the study, breaking the study randomization. In our RCT, the allocation sequence was concealed from all study personnel (except the statistician and study REDCap programmers), including the investigators, field staff, and participants. It was not possible to predict or decipher the next allocation because the sequence was uploaded to REDCap and maintained on the backend so that both key study staff and participants did not have access to the sequence and were blinded to treatment assignment. Step by step instructions on the randomization module configuration are provided in the supplementary materials (Additional file 10: Randomization). We used the User Rights tool to control who can set up and perform the randomization or view the allocation (Additional file 9).



**Additional file 10.**



### Blinding of treatment arm assignment

We used the REDCap User Rights tool to designate which study personnel had access to which aspects of the project and its setup. The highest-level project design and setup privileges were restricted to only a few key study personnel: those responsible for programming, updating, and maintaining the survey. We masked principal investigators from the participants’ groups throughout the study by limiting their User Rights (Additional file 9). Due to the nature of the intervention, participants were aware of their allocated group once they did or did not receive their antibody test results within 12 h. Moreover, because participants self-reported the outcomes, ascertainment of the outcome was not masked.

### Returning test results

The intervention in our RCT was the timing of receiving antibody test results: receiving the results within hours vs. after 4 weeks. We used REDCap functionality to communicate antibody test results to participants in a secure manner, on the timeline dictated by their treatment arm assignment.

REDCap’s Survey Login feature can be helpful when different messages need to be sent to participants depending on their treatment arm or when investigators need to send results to participants. We applied the Survey Login option to a REDCap instrument that contained participants’ antibody test results. Enabling the Survey Login on a REDCap survey will force participants to log in before they can view the survey. In our study, participants made their passwords (survey log-in code) at the baseline survey and used it to log in and view their antibody test results. Moreover, we used the Automated Survey Invitations (ASI) feature to send the result notification emails to participants, with participant-specific login-secured Results Report URL embedded in the email text. ASI also helped us manage the timing of the message delivery based on the participant’s allocated group. ASI allows for the automated sending of an invitation to be triggered by the completion of a previous instrument in addition to other conditions. Thus, the messages regarding the antibody test result report were set to go out to one group 12 h after the completion of the baseline lab visit while, for the other group, they were set to be sent out 4 weeks after the completion of the baseline lab visit. ASI feature is useful for conducting behavioral RCTs where different study arms receive the intervention at different times (Additional file 11: Returning Test Results).



**Additional file 11.**



### Longitudinal study design

#### Events

In longitudinal projects, there are multiple time points that data are collected. In our study, we collected data at baseline survey, baseline laboratory visit, three biweekly follow-up surveys, and an end-line survey (i.e., fourth follow-up survey at the termination of the study). In REDCap, each of these time points for data collection is called an Event. A set of one or more data collection instruments can be used for each Event. To make our longitudinal project we first added our Events to REDCap and next designated our Event-specific data collection instruments to appropriate Events (Additional file 12: Longitudinal Study Design).



**Additional file 12.**



#### Follow-up surveys

Four follow-up surveys were designed to be administered every 2 weeks after the baseline laboratory visit. We decided to use surveys instead of face-to-face interviews because the latter is prone to different types of interviewer bias, such as interviewer gender bias [31]; more importantly, face-to-face interviews were not advised in Fall 2020 to minimize the risk of COVID-19 spread. Follow-up surveys were originally conceptualized as separate Events within the structure of the original longitudinal REDCap project. However, we encountered an impediment to the use of these as separate events. We had imported sampled students’ names and email addresses to our REDCap project. However, only a portion of the sampled students enrolled in the study. We needed to send the follow-up surveys only to this portion of students, and not the whole sample. Unfortunately, REDCap Survey Distribution Tools, the main setting for sending out study invitations, does not have an option to send conditional, automated email invitations. Thus, instead of using the longitudinal design within REDCap, we created four additional separate projects. The follow-up survey invitations were sent on Mondays and an automated reminder was sent the following Thursday of the same week. We merged all follow-up survey data with the baseline and laboratory test results by study identifier to create the final longitudinal dataset for analysis. We suggest investigators test all the interacting components of a project completely and multiple times before moving it to production mode and actual data collection to ensure all project pieces are working as expected.

## Discussion

In this study, we reported how we used different tools and features within REDCap to complete various parts of a large randomized controlled trial, including sending study invitations, eligibility screening, consenting procedures, lab visit appointment and reminders, data collection and maintenance of data confidentiality, randomization, blinding, returning test results, and follow-up surveys.

Not many other methodological studies have been published on how to implement REDCap when conducting scientific studies. Lawrence et al. introduced an eConsent framework in REDCap [14]; Crane et al. reported how they used Application Programming Interface (API), an interface that enables external applications connect to REDCap, for data management in their web-based therapeutic intervention [32]; and Blumenberg and Barros reported how they used REDCap for schedule assessments, data collection, and other study procedures in their prospective cohort study [16]. Our study adds to this literature by providing a practical example on how to use REDCap when completing different stages of a large RCT.

Similarly, limited studies have previously used the REDCap randomization module in their RCT studies, assessing interventions on diabetes control [33] and smoking cessation [34, 35]. However, the randomization module could be useful in RCTs that address different health topics, particularly in RCTs that aim to assess the effectiveness of psychosocial or behavioral treatments, including the growing field of ecological momentary interventions (EMI) in behavioral medicine [36]. Investigators can make use of this module along with the mobile friendly REDCap instruments to conduct their RCTs on EMIs.

### Recommendations for REDCap developers

Given the fast-moving and changeable nature of RCTs, we suggest that REDCap continues to innovate and provide increased flexibility and automation for administering such studies. Specifically, we suggest REDCap 1) add a functionality whereby the system displays a warning before sending a survey invitation email if the subject line is missing, 2) add an option in Survey Distribution Tools to send conditional, automated email invitations, 3) provide the option of selecting data structure (Long vs. Wide) when exporting the data to minimize the data processing efforts, 4) add an automated functionality so participants can unsubscribe from a REDCap project and investigators can keep track of the unsubscriptions, and 5) add the am-pm time validation format as this is the common time format in the U.S. Lastly, through its institutional instances, we suggest REDCap publicizes its capabilities for conducting RCTs.

## Conclusions

REDCap is a widely available data collection system that offers powerful tools for longitudinal data collection, reduction of biases within studies, and the overall implementation and coordination of RCTs. Investigators can make use of this electronic data capturing system to successfully complete their RCTs.

## Supplementary Information

**Additional file 1.**

## Data Availability

The metadata of our entire REDCap project are included in this published article as Additional File 1. The individual level data collected/or analysed during the RCT study are not publicly available due to protection of participants privacy and confidentiality but are available from the corresponding author on reasonable request.

## Abbreviations

REDCap: Research electronic data capture
RCT: Randomized controlled trial
PHI: Protected health information
